# Evaluation of the Findings of Patients Who Underwent Sutureless Flanged Transconjunctival Intrascleral Intraocular Lens Implantation with or without Pars Plana Vitrectomy

**DOI:** 10.1155/2021/4617583

**Published:** 2021-09-01

**Authors:** Elcin Suren, Mustafa Kalayci, Ersan Cetinkaya, Mehmet Fatih Kucuk, Mehmet Erkan Dogan, Doğan Durmaz, Fulya Duman, Muhammet Kazim Erol

**Affiliations:** ^1^University of Health Sciences, Antalya Training and Research Hospital, Department of Ophthalmology, Antalya, Turkey; ^2^Akdeniz University Hospital, Department of Ophthalmology, Antalya, Turkey

## Abstract

**Purpose:**

To compare the visual outcomes and complications of patients who underwent flanged transconjunctival sutureless intrascleral intraocular lens (SIS IOL) implantation after anterior and pars plana vitrectomy.

**Methods:**

All patients who underwent flanged transconjunctival SIS IOL fixation using a 27-gauge needle between September 2017 and November 2019 and were followed up for at least six months were evaluated. The cases in which anterior vitrectomy was performed were classified as Group 1, and those that underwent pars plana vitrectomy were classified as Group 2. The best-corrected visual acuity (BCVA), spherical equivalent values, corneal endothelial cell density, and intraocular pressures were compared between the two groups before and after the operation. Intraoperative and postoperative complications were assessed.

**Results:**

The study included 108 eyes of 108 patients who were included in the study. Group 1 consisted of 48 patients and Group 2 comprised of 60 patients. When the findings between Groups 1 and 2 were compared in the postoperative period, there was no statistically significant difference in terms of the mean intraocular pressure increase, endothelial cell density, BCVA, and spherical equivalent value (*P*=0.818, 0.601, 0.368, and 0.675, respectively). When all the patients were considered as a single group, the mean spherical value at the sixth postoperative month was 0.3 ± 2.2 D (min-max, (−5.5)–(+6)), the mean cylindrical value was −1.7 ± 2.4 D (min-max, (−9.25)–(+4)), and the mean spherical equivalent value was −0.5 ± 2.3 D (min-max, (−6.5)–(+6)).

**Conclusion:**

The flanged transconjunctival SIS IOL fixation technique performed using a 27-gauge needle is safe and effective in the patient group with aphakia and lens/IOL dislocation or subluxation. However, in patients planned to undergo flanged transconjunctival SIS IOL implantation, pars plana vitrectomy seems to be a more suitable option than anterior vitrectomy to reduce complications.

## 1. Introduction

The ideal location of the intraocular lens (IOL) is inside the capsular bag where the crystalline lens is located [1]. Today, during cataract surgery, IOL is inserted into the capsular bag after cataract extraction. However, in cases where capsule support is insufficient secondary to pseudoexfoliation, trauma, Marfan syndrome, and complicated cataract surgery, there is a need to identify an alternative intraocular area to place the IOL [2]. These options include anterior chamber, iris, and scleral fixations of IOL [3]. Although iris-fixed IOLs fixed to the anterior surface of the iris using a claw-shaped haptic device have been widely used in the past for the correction of aphakia, they are no longer recommended due to their high complication rates and suboptimal visual outcomes [4]. However, both the anterior and posterior iris-claw IOLs have undergone significant design changes, including vault modifications [4].

Since the introduction of the IOL fixation technique by creating an intrascleral tunnel without requiring sutures by Gabor in 2007, surgeons have focused on scleral-fixated IOL techniques that do not require sutures [5]. In the same year, Agarwal et al. developed a sutureless technique in an attempt to prevent IOL dislocation by applying fibrin glue to the tip of the intrasclerally placed haptics [6]. However, neither Gabor using a 24-gauge needle nor Agarwal et al. using a 23-gauge needle to perform these operations were successful in preventing the postoperative hypotonia risk.

The introduction of sutureless intrascleral fixation of IOL by Yamane et al. using a 27-gauge needle in 2014 has led to this technique becoming increasingly popular worldwide [7]. Furthermore, in 2017, Yamane et al. further developed this technique by flanging the tip of the intrasclerally placed haptics through low-temperature cautery, thus solving the problem of haptic dislocation in IOL. The Yamane technique has since attracted increasing interest of ophthalmic surgeons since it does not cause conjunctival scars because of the absence of conjunctival dissection, nor does it require the use of tunnel or fibrin glue; furthermore, the tilt rate of IOLs fixed with this technique is similar to that of in-the-bag IOLs [8].

Yamane et al. applied the flanged sutureless intrascleral (SIS) IOL technique using a 30-gauge needle, but this type of needle may not be easily available in many countries. In addition, although in the technique, vitreous cleaning was undertaken by anterior vitrectomy or pars plana vitrectomy (PPV), there is currently no study in the literature that evaluates the effect of these two operations on flanged transconjunctival SIS IOL fixation. In this study, we aimed to evaluate the results of this technique using 27-gauge needles after anterior vitrectomy and PPV.

## 2. Methods

All patients who underwent flanged transconjunctival SIS IOL fixation using 27-gauge needles between September 2017 and November 2019 were examined. All operations were performed by a single surgeon (M.K.E.) at Antalya Training and Research Hospital using the same method. The study protocol was approved by the Ethics Committee of Antalya Training and Research Hospital. All clinical procedures were carried out in accordance with the principles of the Declaration of Helsinki. Written informed consent was obtained from all patients.

Basic demographic data, such as age, gender, and the operated eye, were recorded. The patients that underwent SIS IOL fixation after anterior vitrectomy were classified as Group 1 and those that underwent SIS IOL fixation after PPV were classified as Group 2. All patients were followed up for at least six months. The best-corrected visual acuity (BCVA) evaluation, slit-lamp examination, intraocular pressure (IOP) measurement with a Goldmann applanation tonometer, and dilated fundus examination of all patients were undertaken at all clinic visits before and after surgery, and the results were recorded. Corneal endothelial cell density was measured with a specular microscope (Tomey EM-3000, Nagoya, Aichi, Japan) at six months before and after surgery. Patients with visually significant pathologies, such as corneal scar, retinal detachment, epiretinal membrane, macular edema, and glaucomatous optic atrophy, and those that were followed up for less than six months were excluded from the study. In addition, pediatric cases and those with trauma endophthalmitis were excluded from the study.

Optical coherence tomography (OCT) (Zeiss Cirrus 5000 HD-OCT, Carl Zeiss Meditec, Dublin, CA, USA) was performed preoperatively in patients with clear media and at the first, third, and sixth months postoperatively in all patients. Optical biometry was undertaken using a Lenstar LS 900 device (Haag-Streit, Switzerland), and the SRK-*T* formula was used for the IOL power calculations. Immersion biometry was used in cases where the measurements could not be obtained by optical biometry. The value found by subtracting 0.50 diopters from the measurement value obtained from all patients was determined as the target value.

In patients that developed nucleus drop or posterior capsule rent, anterior vitrectomy or PPV was performed according to the surgeon's preference when the complication developed, and then flanged transconjunctival SIS IOL fixation was undertaken, with the whole operation being completed in one session. In patients who were referred to or presented to our clinic from an external center (those with aphakia, subluxated or dislocated cataracts, and dislocated IOLs), flanged transconjunctival SIS IOL fixation was performed following anterior vitrectomy or PPV in a single session. Eyecryl Plus monofocal three-part intraocular lenses were used in all patients (Biotech, Luzern, Switzerland).

Complications, such as postoperative hyphema, transient corneal edema, endophthalmitis, increased IOP, postoperative hypotonia, vitreous hemorrhage, retinal detachment, haptic erosion, optic capture, macular edema, and reverse pupillary block (RPB), were recorded. Hypotonia was defined as an IOP of ≤5 mmgHg.

### 2.1. Surgical Procedure

The patients were placed under retrobulbar anesthesia, and the surgery was performed from conjunctival entry points 2 mm behind the limbus at 3 o'clock and 9 o'clock positions with a 180-degree angle in between. Of the patients included in the study, 60 underwent PPV and 40 underwent anterior vitrectomy. Following vitrectomy, after a limbal incision, a three-piece IOL placed in a cartridge was implanted using an injector, with the anterior haptic and optic part being in the anterior chamber and the posterior haptic left outside the limbal incision. A 27-gauge needle was inserted into the sclera from the conjunctival entry point and advanced to the posterior, parallel to the iris, forming an angle. Using serrated jaws microforceps (D.O.R.C. International, Zuidland, the Netherlands) with a 23-gauge needle, the anterior haptic of a three-piece IOL was inserted from the side parasynthesis to the needle end seen behind the iris in the anterior chamber, and the needle was advanced through the lumen. Then, it was removed from the sclera and conjunctiva from the needle entry point. The anterior haptic was held, and the end of the haptic was cauterized using an ophthalmic cautery device (Accu-Temp Cautery, Beaver-Visitec International, Inc., Waltham, USA) to flange the end of the haptic at a diameter of 0.3 mm. The flanged end was implanted intrasclerally, as described by Yamane et al. The same technique was applied at a 180-degree opposite angle to the following haptic for intrascleral fixation. Peripheral iridotomy was performed using a vitrectomy cutter to prevent the iris capture of IOL formation after myosis. The viscoelastic substance was aspirated, and the anterior chamber was formed using a balanced salt solution. In patients who underwent pars plana vitrectomy, the cannula was removed, and wound integrity was provided at the end of the procedure.

In addition, IOL fixation was not preferred if the dislocated lens was a monofocal three-piece IOL.

### 2.2. Statistical Analysis

The Statistical Package for the Social Sciences (SPSS v. 23.0, Chicago, USA) was used for the statistical analyses. Descriptive statistics, i.e., mean ± standard deviation (SD) values, were used to describe quantitative data, and frequencies and percentages were used for qualitative data. Preoperative and postoperative data were analyzed using the paired *t*-test. Student's *t*-test was conducted to compare the data between the two groups. *P* < 0.05 was considered statistically significant. Visual acuity was converted to the logarithm (logMAR) of the minimum resolution angle for the analysis.

## 3. Results

The mean follow-up time was 15.2 ± 6.3 months in Group 1 and 13.8 ± 6.8 months in Group 2. There was no statistically significant difference between the two groups in terms of the follow-up time (*P*=0.282). The mean age of the patients was 63.1 ± 10.7 years in Group 1 and 61.9 ± 8.8 years in Group 2, indicating no significant difference (*P*=0.534). Indications for surgery were dislocated posterior chamber IOL in 52 patients (48.2%), aphakia in 39 patients (36.1%), and crystalline lens subluxation in 17 patients (15.7%). Patient characteristics are shown in Table 1.

Considering all the patients as a single group, the mean preoperative BCVA was 2.1 ± 0.8 logMAR, while the mean postoperative sixth-month control BCVA was 0.5 ± 0.4 logMAR, indicating a statistically significant difference between the two measurements (*P* < 0.001). The postoperative first-month and sixth-month BCVA results were 0.8 ± 0.5 and 0.5 ± 0.4 logMAR, respectively, and the increase in visual acuity was significant (*P* < 0.001). The mean preoperative corneal endothelial cell density was 2535 ± 388 cells/mm^2^, and this value was found to decrease to 2260 ± 358 cells/mm^2^ at the postoperative sixth month, resulting in a statistically significant difference (*P* < 0.001). At the postoperative sixth month, the mean spherical value was 0.3 ± 2.2 D (min-max, (−5.5)–(+6)), the mean of the cylindrical value was −1.7 ± 2.4 D (min-max, (−9.25)–(+4)), and the mean spherical equivalent value was −0.5 ± 2.3 D (min-max, (−6.5)–(+6)).

As shown in Table 2, there was no statistically significant difference between Groups 1 and 2 in terms of the mean postoperative IOP increase, endothelial cell density, BCVA, and spherical equivalent value (*P*=0.818, 0.601, 0.368, and 0.675, respectively).

### 3.1. Complications

The distribution of intraoperative and postoperative complications is shown in Table 3. Intraoperatively, three patients in Group 1 and one patient in Group 2 developed retinal breaks due to the 27-gauge needle entry. Cases with a retinal break were successfully given laser treatment. Furthermore, two patients in Group 2 developed hemorrhage at the sclerotomy site, and two patients in Group 1 developed small choroidal hemorrhage.

Early postoperative complications were defined as those that developed within the first month after surgery. An IOP increase was observed in five patients in Group 1 and one patient in Group 2. A topical anti-glaucomatous agent was given to all of these patients, and they responded to this treatment. Two of the patients with increased IOP continued anti-glaucomatous treatment after the first month. Hypotonia was observed in two patients in Group 1 and three patients in Group 2 within the first week of surgery. There was no need for a second intervention in these patients, and it was observed that hypotonia improved in their follow-up. Vitreous hemorrhage was observed in two patients in each group and resolved during the follow-up. In Group 1, two patients had retinal detachment during their second-week follow-up. Both underwent pars plana vitrectomy and 360-degree retinal laser treatment within the same week. Dislocation was observed on one side of the intrascleral haptics in one patient in each group. A haptic correction operation was performed in these patients on the same day, and no problem was observed in their follow-up. Transient corneal edema was not observed in Group 1 but was present in two patients in Group 2.

One patient in each group who had macular edema due to late postoperative complications was prescribed topical nepafenac drops four times a day. In the follow-up, macular edema was improved in both patients. In our study, the development of RPB, uveal tissue inflammation, or endophthalmitis was not recorded.

## 4. Discussion

According to our review of the literature, this study evaluated the largest number of cases to date in terms of the outcomes of the flanged transconjunctival SIS IOL fixation and provided important data from an average follow-up period exceeding 12 months with no short-term follow-up, comparing the results of two groups undergoing PPV and anterior vitrectomy.

The flanged transconjunctival SIS fixation technique of the haptic of a three-piece IOL described by Yamane et al. [8] has many advantages over sutured SIS IOL fixation techniques reported by Gabor [5] and Agarwal [6]. Advantages are that there is no need for an intrascleral pouch, adhesive use, such as fibrin glue, or conjunctival dissection. In addition, another advantage can be considered as the much lower risk of hypotonia in this technique that allows for the use of 27- and 30-gauge needles compared to 23- and 24-gauge needles required by the other techniques. In all cases in our study, we used 27-gauge needles due to the problems in the accessibility of 30-gauge needles in Turkey. We observed hypotonia at a rate of 4.6% within the first week of the operation. All hypotonic eyes were observed normotonically in the following week. Abbey et al. [9] reported the rate of hypotonia as 13.3%. In another study conducted in 2019, Czajka et al. [10] determined the rate of postoperative hypotonia as 19.4%. In that study, the authors compared SIS IOL fixation techniques with and without the use of trocar and stated that hypotonia was more common in the trocar group. In the current study, the rate of postoperative hypotonia being higher in patients in Group 2 suggests that 25-gauge trocars used for the PPV entry may increase the development of this complication. The use of a smaller-gauge trocar for PPV can significantly reduce postoperative hypotonia.

In this study, the most frequently observed postoperative complication was the increase in IOP (5.6%), which we successfully resolved with topical treatment in all cases. Yamane et al. [8] determined the rate of increased IOP as 2%. In a 2018 study, Stem et al. [3] reported the rate of increased IOP as 23%, which they attributed to RPB developing postoperatively, and recommended performing intraoperative prophylactic iridotomy in these patients. RPB is a rare finding after the scleral fixation of IOL, but it may result in pigment dispersion or iris capture and therefore might require treatment with postoperative laser peripheral iridotomy to prevent these negative effects [11, 12]. In our study, RPB was not observed in any of the patients as a result of intraoperative prophylactic iridotomy being performed in all patients. Thus, our rate of increased IOP was also much smaller compared to the study of Stem et al. [3] Another important outcome of performing prophylactic iridotomy in our study was that no pigment dispersion occurred in any patient. However, the majority of our patients who had increased IOP postoperatively were in Group 1 (83.3%). In patients undergoing anterior vitrectomy, viscoelastic substance possibly remaining after the insufficient washing of the anterior chamber may have caused a temporary IOP increase.

IOL haptic dislocation being observed among the first cases operated in each group led us to consider two different reasons: the lack of complete flanging due to inadequate cauterization in the early stages of the learning curve and the vertical aspect being more than normal due to the insertion of the needle inside the sclera to the posterior of the iris without creating a complete parallel area of 2 mm from the intrascleral entry point. Although according to Czajka et al. [10], IOL haptic dislocation might be due to gas tamponade used in PPV and extra sutures might be required in the intrascleral region in which the haptic is placed, the absence of IOL dislocation in the later stages of our study does not support this idea. We consider that haptic dislocations can be prevented by sufficient flanging and forming intrascleral entry points 2 mm posterior to the limbus, running first parallel inside the sclera and then parallel to the iris with a 90-degree inclination to the entry point. In addition, when postoperative optical biometry measurements were reassessed in two patients with haptic dislocation, the diameter of the cornea was 12 mm in both eyes, which also suggests that there may be another reason for dislocation. In their 2015 study, Jacob et al. [13] stated that IOL should be placed vertically in eyes with a corneal diameter larger than 11.5 mm. In a more recent study, Czajka et al. [10] noted that the frequency of haptic dislocation increased in eyes with a corneal diameter greater than 12 mm. From this perspective, we think that the preoperative corneal diameter should be measured in patients planned to undergo sutureless flanged SIS IOL fixation, and if this diameter is above 12 mm, haptic entries should be at 6 and 12 o'clock positions.

In studies where flanged transconjunctival SIS IOL fixation was performed and the endothelial cell density was evaluated before and after surgery, Yamane et al. [8] found a significant decrease in endothelial cell density, while Kelkar et al. [14] reported this difference as not significant. In our study, a significant decrease was observed in the corneal endothelial cell density in the postoperative period compared to the preoperative period, which is consistent with the findings of Yamane et al. However, when we compared the postoperative endothelial cell density between Groups 1 and 2, there was no significant difference.

Mora et al. [4] performed a comparative analysis of the safety and functional results of anterior and retropupillary iris-claw IOL fixation in their study published in 2018. In this study, BCDVA significantly improved after surgery in both groups, without significant difference between the two groups. Again, according to the results of this study, compared with the preoperative assessments, the endothelial cell counts were significantly reduced in both groups after surgery, without a significant intergroup difference [4]. The increase in postoperative visual acuity and decrease in the number of endothelial cells in our study were consistent with the study of Mora et al. In the needle-guided retropupillary fixation of iris-claw IOL technique proposed by Frisina et al., it is noteworthy that despite the increase in postoperative visual acuity, the postoperative endothelial cell density did not decrease [15]. However, the relatively high cost of iris-claw IOLs and the difficulty in supplying these lenses to patients forced us to choose the sutureless flanged transconjunctival scleral fixation technique.

In our study, the incidence of postoperative retinal detachment was 1.9%, which is in agreement with other studies. Retinal detachment is a rare complication after SIS IOL fixation, and its frequency varies between 0% and 3.8% [10, 16, 17]. Two retinal detachments occurred postoperatively among the first 20 cases in our study, suggesting that surgeons should take care in the first stages of the learning curve of the SIS technique. In addition, both cases being in Group 1 indicates that the vitreous base was not properly cleaned at the haptic entry points during anterior vitrectomy, causing retinal detachment by creating a stretching force between IOL and the vitreous body. Therefore, in patients who only underwent anterior vitrectomy before flanged transconjunctival SIS IOL fixation, complete intraoperative cleaning of the vitreous body in the scleral entry areas may prevent the development of postoperative retinal detachment.

The development of cystoid macular edema after flanged SIS IOL implantation has been reported with very variable rates in different studies. Stem et al. [3] determined the frequency of macular edema as 21% and achieved response to treatment in seven of 11 patients. Yamane et al. [8] found that 1% of cases developed cystoid macular edema. In our study, the incidence of cystoid macular edema was 1.9%. Topical non-steroidal anti-inflammatory drops were started in both patients, and it was observed that edema was resolved during the follow-up. Khan et al. [18], who used Gore-Tex sutures, calculated the incidence of cystoid macular edema as 4.8% while Yeung et al. [19], using 10/0 nylon sutures, reported this rate to be 8%. Based on these results, the use of sutures in the scleral fixation of IOL implants seems to increase the frequency of cystoid macular edema. However, when you have more than one surgical procedure, you have a higher risk of developing CME due to inflammation of the choroid and retina and breakage of the blood-retinal barrier [20]. Postoperative CME may result from an inflammatory process due to disruption of the blood-retina barrier, similar to what is known as the “Irvine–Gass syndrome” that occurs after any intraocular surgery [20].

In this study, the total postoperative spherical equivalent value of all patients was −0.5 ± 2.3 diopters. In addition, when the two groups were evaluated separately, although there was a higher myopic shift in Group 2 than in Group 1, the difference was not statistically significant. In our study, the causes of myopic shift may be the anterior location of IOL due to its short diameter and excessive cauterization of the haptic tip during flanging. Yamane et al. [8] compared four different types of IOL in their study and observed myopic shift in three IOLs and hyperopic shift in one IOL. However, there are no studies comparing the spherical equivalent value of patients that have undergone anterior vitrectomy to those having undergone pars plana vitrectomy before flanged transconjunctival SIS IOL fixation.

The limitations of our study include its retrospective nature and the lack of an evaluation of the IOL tilt level. However, our study also had certain strengths, such as the number of patients evaluated, the mean follow-up time exceeding 12 months in both groups, and being the first to compare the results of the flanged transconjunctival SIS IOL technique between two vitrectomy groups.

In conclusion, according to our clinical observation and the results of this study, the flanged transconjunctival SIS IOL fixation technique using a 27-gauge needle is safe and effective in patient groups with aphakia and lens/IOL dislocation or subluxation. However, in patients planned to undergo flanged transconjunctival SIS IOL implantation, performing pars plana vitrectomy seems to be a more suitable option than anterior vitrectomy to reduce complications.

## Figures and Tables

**Table 1:** Patient characteristics.

Characteristics	Group 1	Group 2	*P* value
Number of eyes	40	68	
Age (years, mean ± SD)	63.1 ± 10.7	61.9 ± 8.8	0.534
Gender (male/female)	23/17	36/32	

*Diagnosis*			
Dislocated PC IOL	7	15	
Subluxated PC IOL	6	10	
Opacified PC IOL	2	2	
Aphakia	18	21	
Subluxated cataract	3	11	
Dislocated nucleus	4	9	

*Follow-up (months)*			
Mean ± SD	15.2 ± 6.3	13.8 ± 6.8	0.282
Range	6–36	6–36	

SD, standard deviation; PC, posterior chamber; IOL, intraocular lens.

**Table 2:** Comparison of parameters between Groups 1 and 2.

Parameter	Group 1	Group 2	*P* value
Mean postoperative IOP (mmHg)	16.3 ± 5.9	16.8 ± 13.1	0.818
Mean postoperative endothelial cell count (cells/mm^2^)	2390 ± 389	2430 ± 368	0.601
Mean postoperative BCVA (logMAR)	0.63 ± 0.40	0.56 ± 0.43	0.368
Mean postoperative spherical equivalent (diopters)	−0.3 ± 2.3	−0.5 ± 2.3	0.675

IOP, intraocular pressure; BCVA, best-corrected visual acuity.

**Table 3:** Intraoperative, early, and late complications of the two groups.

	Group 1	Group 2
*Intraoperative*		
Retinal break (iatrogenic)	3	1
Hemorrhage from sclerotomy	0	2
Small choroidal hemorrhage	2	0

*Early*		
Increased IOP (>25 mmHg)	5	1
Hypotony (IOP ≤ 5 mmHg)	2	3
Vitreous hemorrhage	2	2
Transient corneal edema	0	2
Retinal detachment	2	0
IOL dislocation	1	1

*Late*		
Increased IOP	2	0
Cystoid macular edema	1	1
Iris capture of IOL	1	1

IOP, intraocular pressure; IOL, intraocular lens.

## Data Availability

The dataset used for analyses may be requested from the corresponding author for use in scholarly work related to the field.
